# Detection of Dendritic Spines Using Wavelet-Based Conditional Symmetric Analysis and Regularized Morphological Shared-Weight Neural Networks

**DOI:** 10.1155/2015/454076

**Published:** 2015-11-24

**Authors:** Shuihua Wang, Mengmeng Chen, Yang Li, Yudong Zhang, Liangxiu Han, Jane Wu, Sidan Du

**Affiliations:** ^1^Department of Electronic Engineering, Nanjing University, Nanjing 210024, China; ^2^School of Computer Science and Technology, Nanjing Normal University, Nanjing 210023, China; ^3^State Key Laboratory of Brain and Cognitive Science, Institute of Biophysics, Chinese Academy of Sciences, Beijing 100101, China; ^4^Department of Neurology, Lurie Cancer Center, Center for Genetic Medicine, Northwestern University School of Medicine, Chicago, IL 60611, USA; ^5^University of Chinese Academy of Sciences, Beijing 100101, China; ^6^Translational Imaging Division, Columbia University, New York, NY 10032, USA; ^7^School of Computing, Mathematics and Digital Technology, Manchester Metropolitan University, Manchester M1 5GD, UK

## Abstract

Identification and detection of dendritic spines in neuron images are of high interest in diagnosis and treatment of neurological and psychiatric disorders (e.g., Alzheimer's disease, Parkinson's diseases, and autism). In this paper, we have proposed a novel automatic approach using wavelet-based conditional symmetric analysis and regularized morphological shared-weight neural networks (RMSNN) for dendritic spine identification involving the following steps: backbone extraction, localization of dendritic spines, and classification. First, a new algorithm based on wavelet transform and conditional symmetric analysis has been developed to extract backbone and locate the dendrite boundary. Then, the RMSNN has been proposed to classify the spines into three predefined categories (mushroom, thin, and stubby). We have compared our proposed approach against the existing methods. The experimental result demonstrates that the proposed approach can accurately locate the dendrite and accurately classify the spines into three categories with the accuracy of 99.1% for “mushroom” spines, 97.6% for “stubby” spines, and 98.6% for “thin” spines.

## 1. Introduction

Dendritic spines are small “doorknob” shaped extensions from neuron's dendrites, which can number thousands to a single neuron. Spines are typically classified into three types based on the shape information: mushroom, stubby, and thin. “Mushroom” spine has a bulbous head with a thin neck; “stubby” spine only has a bulbous head; “thin” spine has a long thin neck with a small head. Research has shown that the changes in shape, length, and size of dendritic spines are closely linked with neurological and psychiatric disorders, such as attention-deficit hyperactivity disorder (ADHD), autism, intellectual disability, Alzheimer's disease, and Parkinson's disease [1–5]. Therefore, the morphology analysis and identification of structure of dendritic spines are critical for diagnosis and further treatment of these diseases [6, 7].

Traditional manual detection approach of dendritic spines detection is costly and time consuming and prone to error due to human subjectiveness. With the recent advances in biomedical imaging, computer-aided semiautomatic or automatic approaches to detect dendritic spines based on image analysis have shown the efficacy. SynD method proposed by Schmitz et al. [8] is a semiautomatic image analysis routine to analyze dendrite and synapse characteristics in immune-fluorescence images. For the fluorescence imaging, the neurite and soma were captured in the separated imaging channels. In that case, soma and synapse were detected without intervention from neurite [9–11] based on the channel information. However, this method cannot be extended to the images, of which the information is captured in the same channel. Therefore, many other methods were proposed to solve this problem, for instance, ImageJ [12], NeuronStudio [13], NeuronJ [14], and NeuronIQ [15]. However, these methods have some limitations. For example, NeuronIQ was designed for the confocal multiphoton laser scanning. NeuronJ was used to trace the dendrite growing in the condition of manually marking the dendrite first. Koh et al. detected spines from stacks of image data obtained by laser scanning microscopy [16]. The algorithm first extracted the dendrite backbone defined as the medial axis and then geometric information was employed to detect the attached and detached spines according to the shape of each candidate spine region. Features including spine length, volume, density, and shape for static and time-lapse images of hippocampal pyramidal neurons were used as key points for the detection. The disadvantage of this method is that it might lose many spines during the detection because of the thresholding method used in this case. To overcome this problem, Xu et al. proposed a new detection algorithm for the attached spines from the dendrites by two grassfire steps [17]: a global threshold was chosen to segment the image and then the medial axis transform (MAT) was applied to find the centerlines of the dendrites. Then some large spines (noncenterlines) were removed from the centerlines. After the backbone was extracted, two grassfire procedures were applied to separate the spine and dendrite. The results of the proposed method were similar to the results of the manual method. Cheng et al. proposed a method using an adaptive threshold based on the local contrast to determine the foreground, containing the spine and dendrite, and detect attached and detached spines [18]. Fan et al. used the curvilinear structure detector to find the medial axis of the dendrite backbone and spines attached to the backbone [19]. To locate the boundary of dendrite, an adaptive local binary fitting (aLBF) energy level set model was proposed for localization. Zhang et al. extracted the boundaries and the centerlines of the dendrite by estimating the second-order directional derivatives for both the dendritic backbones and spines [20]. Then a classifier based on Linear Discriminate Analysis (LDA) was built to classify the attached spines into true and false types. The accuracy of the algorithm was calculated according to the backbone length, spine number, spine length, and spine density. Janoos et al. used the medial geodesic to extract the centerlines of the dendritic backbone [21]. He et al. proposed a method based on NDE to classify the dendrite and spines [22]. The principle of their method was that spine and dendrite had different shrink rates. Shi et al. proposed a wavelet-based supervised method for classifying 3D dendritic spines from neuron images [23].

Existing work is encouraging. However, the problems remain on how to improve accuracy (e.g., accurate extraction of backbone, accurate detection of attached and detached spines). Different from existing approaches, in this paper, we have proposed new algorithms for efficient detection of dendritic spines using wavelet-based conditional symmetric analysis and regularized morphological shared-weight neural network. Our contributions include the following:

1. A new extraction model for dendrite backbone and its boundary localization using wavelet-based conditional symmetric analysis and pixel intensity difference, which can allow accurate extraction of backbone, the first important step for dendritic spines.
2. A new way for spine detection based on regularized morphological shared-weight neural networks (RMSNN) to efficiently detect spines and classify them into right categories, that is, mushroom, thin, and stubby.

The rest of this paper is organized as follows.  Section 2 describes the proposed methods including wavelet-based conditional symmetry analysis and pixel intensity difference for the dendrite detection and localization and regularized shared-weight neural networks for the spine detection. In Section 3, we have conducted experimental evaluation and demonstrated the effectiveness of the proposed algorithm.  Section 4 discusses the results.  Section 5 concludes the proposed approach and highlights the future work.

## 2. Methods

**Figure 1:**
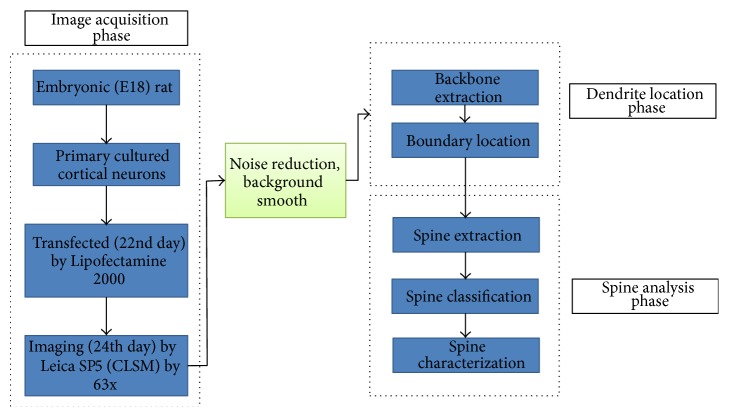
Flowchart of the proposed detection method of the dendritic spines.

Figure 1 shows the steps of our proposed approach to dendritic spines. In the image acquisition phase, we demonstrated the process for the neuron culture, label, and imaging. In the second step, we preprocessed the images by reducing the noise and smoothing the background [24, 25]. Then, we extracted the dendrite backbone based on the conditional symmetric analysis and located the dendrite boundary based on the difference of the pixel intensity. Afterwards, the spines were detected, classified, and characterized by RMSNN.

### 2.1. Image Acquisition

The neurons used for imaging in this paper were cortical neurons, primary cultured from Embryonic 18th- (E18-) day rat and next cultured until the 22nd day in vitro. Then, the neurons were transfected by Lipofectamine 2000 and imaged at the 24th day by Leica SP5 confocal laser scanning microscopy (CLSM) by 63x. The size of the image is 1024 × 1024, and the resolution is 0.24 um/pixel at the confocal layer. The images used for the morphology analysis were obtained by the maximum intensity projection (MIP) of the original 3D image stack. As the images were captured as Z-stack series, we projected the 3D image stack onto the *xy*, *yz*, and *zx* planes, respectively. Since the slices along the optical direction (*z*) provided very limited information and the computation time based on the 3D image stacks is highly increased, it was desired to consider only the 2D projection onto the *xy* plane. The 2D image used for analysis was a maximum intensity projection of the original 3D stack. It was obtained by projecting in the *xy* plane the voxels with maximum intensity values that fall in the way of parallel rays traced from the viewpoint to the plane of projection.

We randomly selected 15 different images from Leica SP5 confocal laser scanning microscopy to form the spines library to test our algorithm. All images contain distinct spines including mushroom, stubby, and thin types. The typical size of the image is 1024 × 1024. Most spines in the images are within a rectangle of 20 × 20 in pixel, but the “thin” spine is within an about 5 × 20 rectangle in pixel. The spines have variable gray-level intensities. Spines collected from the image library were employed to build an image base library. Spine subimages in the library were taken as samples to test the classification accuracy of RMSNN. In order to cover as many cases as possible, the image base library contains distinct sizes and spines with different orientations.

**Figure 2:**
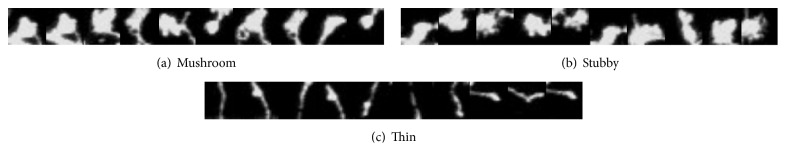
Samples of the subimages used in the image library.

In order to build the golden-standard spine library, five experts in the neuroscience field were employed to manually mark the spines in the collected images and classify the spines into three predefined categories including “mushroom,” “stubby,” and “thin” types. For the conflict of the manual marking, the minority was supposed to be subordinated to the major. Then according to the marked spines, we computed the maximum width, length, area, and the center point. The randomly selected image base library contains about 2700 subimage samples, 900 for each type of spines.  Figure 2 shows some image samples in our image base library. As we can see from the image sample, spines of “mushroom” type contain a thin neck and head, the stubby type connects directly with the dendrite without neck, and the thin type is with the smallest size with only a thin neck and without head.

### 2.2. Image Preprocessing

**Figure 3:**
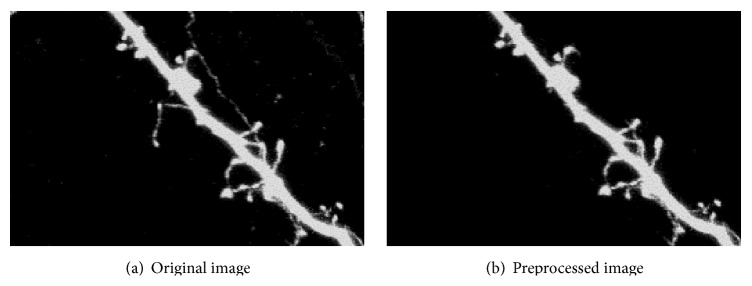
An example of preprocessed image.

Considering the limitation of imaging technique, we have employed the 2D median filter to deal with the noise introduced by the imaging mechanism of the photomultiplier tubes (PMT) and then used the partial differential equation (PDE) proposed by Wang et al. [26] to enhance the image.  Figure 3 shows an example of the original image and the preprocessed result.

### 2.3. Backbone Extraction Using the Wavelet Transformation Based Conditional Symmetric Analysis

Considering the attached spines, it is necessary to firstly locate the dendrites in order to segment the spines from the dendrite. The backbone extraction and boundary localization are critical for dendritic spine classification and analysis, which include the following steps.

**Step 1 .**

Remove the noise and small isolated point-set.

**Step 2 .**

Locate the backbone of the dendrite.

**Step 3 .**

Locate the boundary of the dendrite.

The backbone is defined as the thinning of the dendrite. Due to the variance of width of dendrite, attached and detached spines, it is a challenging task to locate the boundary of the dendrite directly from the preprocessed images. Therefore, we have developed a new extraction model utilizing wavelet transform based conditional symmetric analysis. The essence of this model is to conduct a local conditional symmetry analysis of the contour of the region of interest (ROI) and then compute the center points to produce the backbone of the dendrite.

Due to the complexity of the dendrites and dendrite spines' distribution, we have employed morphological operation to remove the small isolated point-set for the dendrite in the binary image obtained by local Otsu [27–29] via (1), which could decrease the disconnection rate of the dendrite detection:

(1) P=1,more than  n positive pixels in its  3-by-3  window,0,otherwise,

in which *n* is the threshold of the number of positive pixels. The value of *n* could be determined by trial and error method and means that the pixel belongs to the major line if there are more than *n* positive pixels in its 3 × 3 neighborhood window. Otherwise, the value of the pixel is forced to be 0, treated as the small isolated point-set. The determination of the centerline of the dendrite is based on the conditional symmetric analysis.

The symmetric analysis was accomplished via the wavelet transform. We have applied the wavelet transform to detect a pair of contour curves:

(2) φxx,y∂∂xθx,y=ϕ′x2+y2xx2+y2,φyx,y∂∂yθx,y=ϕ′x2+y2yx2+y2,

in which *x* and *y* stand for the coordinate of the contour curve. *φ*_*x*_(*x*, *y*) means the partial derivative of *x* and *φ*_*y*_(*x*, *y*) stands for the partial derivative of *y*, respectively. *θ*(*x*, *y*) is a low pass filter.

For *φ*_*x*_(*x*, *y*) and *φ*_*y*_(*x*, *y*), the scale wavelet transform (WT) could be written as the following equations:

(3) Wx,sfx,yf∗φx,sx,y=s∂∂xf∗θsx,y,Wy,sfx,yf∗φy,sx,y=s∂∂yf∗θsx,y.

Here, *θ*_*s*_ = (1/*s* ^2^)*θ*(*x*/*s*, *y*/*s*). We can get the modulus of the gradient vector as

(4) ∇Wsfx,y=Wx,sfx,yWy,sfx,y,

(5) ∇Wsfx,y=Wx,sfx,y2+Wy,sfx,y2,

(6) Asfx,y=arctan⁡Wy,sfx,yWx,sfx,y,

where ∇ is the gradient vector and the gradient direction is given as (6). The contour points (*x*, *y*) are the local maxima of |∇*W*_*s*_ *f*(*x*, *y*)| in the direction of *A*_*s*_ *f*(*x*, *y*) at scale *s*. However, the local maxima modulus is not the exact edge point.

We selected (7) as the basis function. We set *φ* ^−^(*x*) = −*φ* ^+^(−*x*) and had *φ*(*x*) = *φ* ^+^(*x*) + *φ* ^−^(*x*) as the wavelet function, which had the following properties: gray invariant, slope invariant, width invariant, and symmetric [29, 30]. The advantage is to make the extraction of a pair of contours with accurate protrusions. Consider

(7) φ+=2π4xln⁡1−8x2+21−16x21+1−x29x−8x2+39−16x2−12x1−16x2−39−16x2+81−x2,x∈0,142π4xln⁡8x1+1−x29+39−16x2−12x39−16x2−81−x2,x∈14,342π4xln⁡1+1−x2x−4x1−x2,x∈34,10,x∈1,∞.

The distance between two symmetric points is equal to the scale of the wavelet transform. If the distance between two symmetric points is larger than or equal to the width of regular region, the center point of the symmetric pair can potentially be located outside of the dendrite. The regular region is defined as the dendrite is smooth, where the function has a stable variation along the axis. Thus, we defined the stable symmetry as follows.

If the scale of wavelet transform is larger than or equal to the width of regular region, the modulus maxima points generate two new parallel contours inside the periphery of the dendrite. All the symmetric pairs of the wavelet transforms that do not have a counterpart are defined as the unstable symmetry. In this case, we have considered the width as the constraint condition. In the direction of the perpendicular to the gradient direction, we selected the width nearest to the regular region.

The center of every symmetric pair located on the centerline of the original regular region of the stroke point. Finally, the backbone of the regular region was defined by the curve of all connected symmetric points.

### 2.4. Boundary Location Based on the Pixel Intensity Difference

The morphological operation of removing noise blurred the boundary. Therefore, after localization of backbone, the boundary of the dendrite was detected via varies of the pixel intensity of the preprocessed image from Section 2.2. We can observe that the pixel intensity of the line pixel changes abruptly at the boundary locations. The boundary location was performed in two steps. In the first step, we have searched the image along the two directions perpendicular to the local line direction until the pixel intensity of the line pixel changed sharply. We set a threshold for each pixel. The local line direction is determined as

(8) $$\begin{array}{c} A_{s}f\left(\;x,y\;\right)=\arctan\left(\;\frac{W_{y,s}f\left(x,y\right)}{W_{x,s}f\left(x,y\right)}\;\right). \end{array}$$

The formulation of each pixel is given by (*α*, *I*(*p*)), in which *I*(*p*) is the pixel intensity of point *p* in the original image and *α* is a predefined pixel intensity value, that is,

(9) $$\begin{array}{c} \text{if}\left\{\begin{array}{cc} I\left(p\right)\geq \alpha , & p\text{ belongs to the line pixel}\\ I\left(p\right)<\alpha , & p\text{ does not belong to the line pixel.} \end{array}\right. \end{array}$$

In the second step, some boundary points that were not on the searching path could be missed. The missed boundary points were detected from the neighboring boundary points. Provided that there are two known boundary points, if they are adjacent, there were no other boundary points between them; otherwise, the method proposed by Tang and You [31] was used to find the missed points, which can link the two points into a discrete line with one point as the starting point and the other one as the ending point.

There are several advantages of our proposed algorithms for backbone detection and boundary location. (1) The first are computing efficiency and noise reduction. Our approach uses less computing time than the method based on the derivatives of the Gaussian kernel and is more robust when dealing with the noise. (2) Meanwhile, it reduces the error rate for misclassifying spine pixels as dendrite pixels and sharply reduces the disconnection rate, which means our approach is more robust when dealing with the disturbance information than other methods, such as NDE proposed by He et al. [22].

### 2.5. Spine Detection Based on Regularized Morphological Shared-Weight Neural Network (RMSNN)

**Figure 4:**
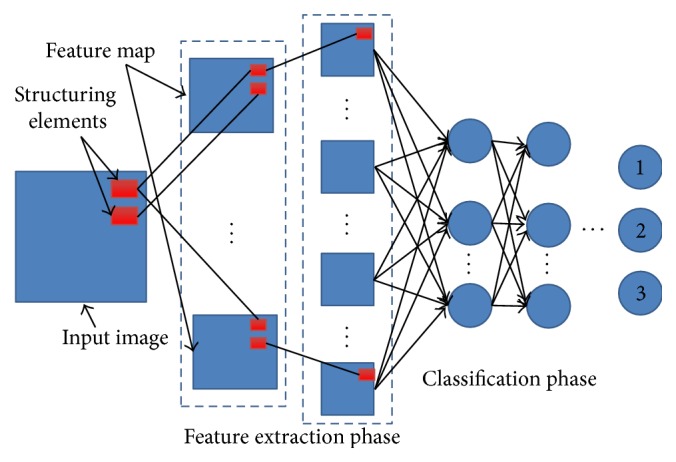
Structure of morphological shared-weight neural network.

Considering the dendritic spine's structure, we have employed the regularized morphological shared-weight neural networks for the detection and classification of spines. The regularized morphological shared-weight neural networks consist of two-phase heterogeneous neural networks in series as shown in Figure 4: the first phase is for feature extraction and the second phase is for classification. In the first phase, it is accomplished via the gray-scale Hit-Miss transform. The feature extraction phase has multiple feature extraction layers. Each layer is composed of one or more feature maps. Each feature map is generated by the Hit-Miss transform with a pair of structure elements (SEs) from the previous layer and is accompanied by a new pair of SEs, in which one is for the erosion and the other one is for the dilation. In the classification stage, it shows a fully connected Feedforward Neural Network (FNN) [32–34]. The input of FNN is the direct output of the feature extraction stage. The output of the classification stage is a three-node layer, in which each node stands for one type of spine. Figure 4 shows the structure of the morphological shared-weight neural network (MSNN) [35]. The MSNN has been widely applied in the following research fields, including laser radar (LADAR), forward-looking infrared (FLIR), synthetic aperture radar, and visual spectrum image. The existing research demonstrates that the MSNN is robust for detection with rotation, image intensity translation, and occlusion variables [36]. In this paper, we have proposed to apply the regularized morphological shared-weight neural network to spine classification.

Dilation is defined as

(10) $$\begin{array}{c} A⊕B=\left\{\;x\;|\;{\left(\;\hat{B}\;\right)}_{x}\cap A\neq \emptyset \;\right\}, \end{array}$$

in which *A* and *B* are sets in *Z* ^2^ and $\hat{B}$ is the reflection of *B*. *∅* is the empty set. Equation (10) is termed the dilation of *A* by SE *B*. Dilation is the reflection of *B* about its origin, then translated by *x*, with the set of all *x*, which allow $\hat{B}$  to intersect *A* with at least one element.

Erosion is defined as (11) or (12) by the duality of the erosion-dilation relationship:

(11) A⊖B=x ∣ Bx⊆A,

(12) A⊖B=Ac⊕B^c,

in which *A* ^*c*^ is defined as the complement of *A*.

Hit-Miss transform is defined as an operation that detects a given pattern in a binary image based on a pair of disjoint structure elements, one for Hit and the other one for Miss. The result of the Hit-Miss transform is a set of positions, where the first SE fits in the foreground of the input image and the second SE misses it completely:

(13) $$\begin{array}{c} A⊗B=\left(\;A⊖X\;\right)\cap \left(\;A^{c}\left(\;W-X\;\right)\;\right), \end{array}$$

in which *X* is a SE that consisted from set *B*, *W* is an enclosing window of *X*, and (*W* − *X*) is the local background of *X*. By supposing *X* as *H*, the Hit SE, and (*W* − *X*) as *M*, the Miss SE, we can get

(14) $$\begin{array}{c} A⊗B=\left(\;A⊖H\;\right)\cap \left(\;A^{c}⊖M\;\right), \end{array}$$

in which *B* = (*H*, *M*) and it can be written as

(15) $$\begin{array}{c} A⊗B=\left(\;A⊖H\;\right)-\left(\;A^{c}⊕\hat{M}\;\right). \end{array}$$

As far as the gray scale is concerned, we assume the image function as *I* = *f*(*x*, *y*), in which *f*(*x*, *y*) was the intensity value of the point (*x*, *y*). Meanwhile, we made the SE *b*(*x*, *y*). The morphological operation can be thought of as a 3D binary set by way of the umbra transform. The umbra of a 3D surface function is defined as

(16) $$\begin{array}{c} U\left(\;f\;\right)=\left\{\;\left(\;x,y,z\;\right)\;|\;\left(\;x,y\;\right)\in D_{f},\;\;z\leq f\left(\;x,y\;\right)\;\right\}, \end{array}$$

where we take *D*_*f*_ as the domain of *f*. Then the gray scale dilation can be defined as

(17) f⊕bs,t=max⁡fs−x,t−y+bx,y ∣ s−x,t−y∈Df;x,y∈Db.

Meanwhile, erosion is defined as

(18) f⊖bs,t=min⁡fs+x,t+y−bx,y ∣ s+x,t+y∈Df;x,y∈Db.

The gray scale erosion measures the minimum gap between the image values *f* and the translated SE values over the domain of *x*. The gray scale dilation is the dual of the erosion and indirectly measures how well the SEs fit above *f*. The Hit-Miss transform measures how a shape *h* fits under *f* using erosion and how a shape *m* fits above *f* via dilation. The high value of Hit-Miss transform means good fit. The gray scale Hit-Miss transform is independent of shifting in gray scale.

#### 2.5.1. The Feature Extraction Phase

There are four elements associated with each layer of feature extraction phase: feature maps, input, and two structure elements. In the first layer, the subimage is used as input, and the last layer's output is the input of the classification stage. In each feature extraction layer, a pair of Hit-Miss SEs is shared within all the feature maps. These SEs are translated as input weights for the feature map nodes in the feature extraction layer.  Table 1 shows the input parameters and output parameters related to the feature extraction phase.

According to the above parameters, we can define the Hit-Miss transform as follows:

(19) netyh=minx∈Dty⁡ax−tyhx,netym=maxx∈Dty⁡ax−tym^,ay=netyh−netym.

Here, net_*y*_^*h*^ stands for the input for Hit operation in node *y* and *h* means the Hit operation. net_*y*_^*m*^ means the net input for the Miss operation in node *y*. *m* and $\hat{m}$ here mean the Miss operation and reflection of *m*, respectively. *a*_*y*_ is the result of Hit-Miss transform performed at node *y*. The learning rule for the Hit and Miss SE is derived based on the gradient decent as

(20) Δtyh=ηδy∂netyh∂tyhx,Δtym^=−ηδy∂netym∂tym^x,

where *η* is the learning rate of the network and *δ*_*y*_ is expressed as

(21) δy=δy=∑δkkwky.

Equation (21) is for the top level or final extraction layer. *δ*_*y*_ for the lower layers of multiple-layer feature extraction is expressed as

(22) δy=δy=∑δkk∂netyh∂ay−∂netym∂ay,

in which *k* is the node in the layer next to the node *y*.

Based on the back-propagation of error from the classification stage with these learning rules, the MSNN learns the optimized SE to extract the features by each set of Hit-Miss transforms. Consider

(23) $$\begin{array}{c} E=\frac{1}{2}\underset{o}{\sum }{\left(\;t_{o}-O_{o}\;\right)}^{2}. \end{array}$$

Here, *t*_*o*_ stands for the target node output and *O*_*o*_ the actual node output:

(24) Oj=fnetj,netj=∑iwjiOi+Δj,

in which *w*_*ji*_ is the connection weight strength to node *j* from node *i* and Δ_*j*_ is the bias output for node *j*. *w*_*ji*_ is typically learned by the back-propagation of error. The update rule of connecting weight for each connection is expressed as follows:

(25) $$\begin{array}{c} \Delta w_{ji}=-\eta \frac{\partial E}{\partial w_{kj}}=\eta \delta_{j}O_{j}. \end{array}$$

For the output layer nodes, *w*_*kj*_ stands for the connection strength to node *k* from node *j*:

(26) $$\begin{array}{c} \delta_{j}=\left(\;t_{j}-O_{j}\;\right)f^{\prime }\left(\;{\text{net}}_{j}\;\right) \end{array}$$

and for the hidden layer nodes,

(27) $$\begin{array}{c} \delta_{j}=f^{\prime }\left(\;{\text{net}}_{j}\;\right)\underset{k}{\sum }\delta_{k}w_{ji}. \end{array}$$

#### 2.5.2. The Classification Phase

The classification phase takes the output directly from the last feature extraction layer as its input. The parameters used for the classification phase are predefined in the feature extraction phase. There are three output nodes for the classification stage of our algorithm, indicating which type of spines the subimage contains.

#### 2.5.3. Acceleration of the MSNN Based on the Regularization

In order to accelerate the learning rate and decrease the learning epochs, we employed the regularization factor. Regularization is used to reduce near-zero connection weight value to zero, therefore reducing the complexity of the network. It is defined as

(28) Rw=Esw+λEcw,Ecw=∑∀w in networkwi/wo21+wi/w02,

where *E*_*s*_(*w*) is the performance measure of the learning algorithm, the total network error, and *E*_*c*_(*w*) is the complexity penalty of the network model. *λ* is the regularization factor. *w*_0_ is a predefined parameter. Meanwhile, research shows that a network with proper SEs produces better result [36]. Therefore, it is essential to choose the suitable SEs. In this paper, according to the average size of spine and the comparison result in Table 3, we defined the SE as a disk with the radius of 4 pixels.

For the training procedure, the RMSNN takes the subimage as the input and makes one output value for each image. For the testing procedure, our proposed algorithm scans the whole ROI and generates an image named the detection plane, which is based on the outputs from the target class nodes.

## 3. Experimental Evaluation

### 3.1. Experiment Design

We have trained neural networks with the back-propagation algorithm. The subimages were submitted to the input nodes of the neural network. The error of the output was propagated through all the connections. The process repeated until the network converged to a stable state with required MSE. When the MSE approximated to a preset value or the maximum epoch was achieved, the algorithm converged and the training would stop. During the training, the RMSNN took each subimage as the input and produced one output value for each of the three categories.  Figure 2(a) shows the samples of subimages containing mushroom type spine.  Figure 2(b) shows the samples of the subimages containing the stubby type, and Figure 2(c) shows the samples of thin type subimage.

In the training step, the subimage samples were input to the network sequentially. The median-squared error was employed to measure the training effectiveness. For each subimage, the RMSNN produced one output value, which indicated the type of spine in the subimage. Then, we scanned the entire microscopy image and finally generated a detection plane according to the output nodes of RMSNN.

In order to test the classification accuracy, we randomly selected 900 samples for each type of spine, respectively. Following common convention and ease of stratified cross validation, 10 × 10-fold stratified cross validation (CV) was used for the dataset to perform an unbiased statistical analysis. The RMSNN was constructed in the form as two feature extraction layers, one hidden layer with ten hidden neurons and one output layer with three neurons. The input subimage size was 20 by 20 pixels, and the size of the structure elements was with the radius of 4 pixels. The initial weight was in the range of [−1.0,1.0]. The learning rate was set to 0.0015. The maximum training epoch was predefined as 15000. The expected output values for mushroom, stubby, and thin type spines were [100], [010], and [001].

### 3.2. Experiment Results

#### 3.2.1. Backbone Extraction

**Figure 5:**
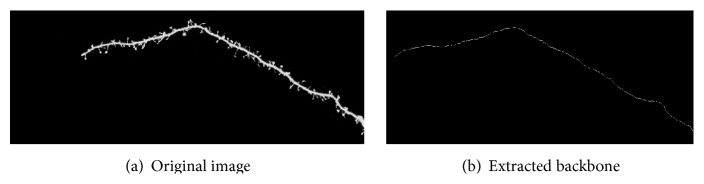
Backbone extraction result.

The extraction result is shown in Figure 5.  Figure 5(a) shows the original image.  Figure 5(b) shows the extracted backbone, of which the width covers merely one pixel.

#### 3.2.2. Boundary Location

**Figure 6:**
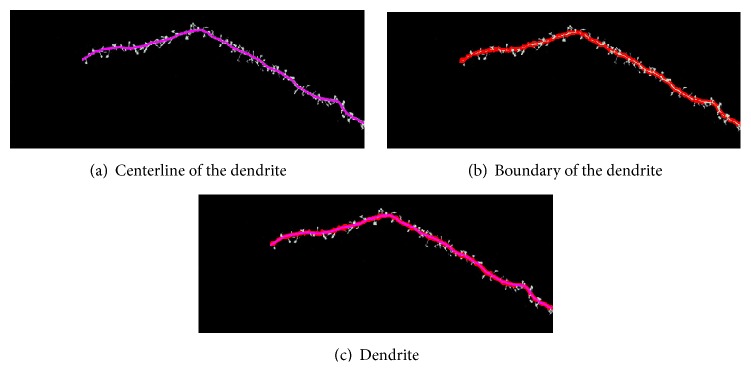
Dendrite location results.

Figure 6(a) shows the mark of the located backbone of the dendrite based on the original image, and Figure 6(b) shows the marked boundary of the dendrite after the backbone is extracted.  Figure 6(c) shows the marked dendrite that determines the starting point of the spine.

#### 3.2.3. Spine Analysis

**Figure 7:**
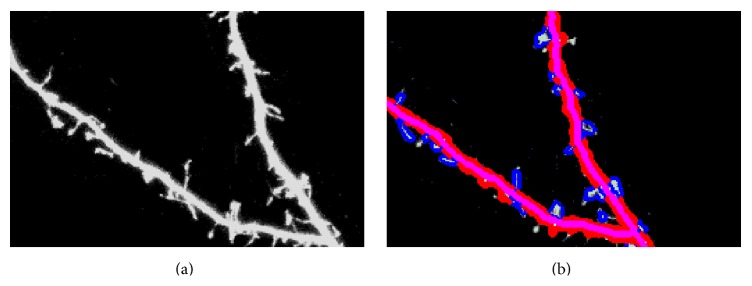
(a) ROI of the original Image. (b) Detection result of the spines.

Figure 7 shows a ROI of our sample image, and Figure 7(b) shows the detection result of the spines. The backbone is marked by the purple color and the boundary is marked by the red color. The spines are marked by their periphery of blue color.

**Figure 8:**
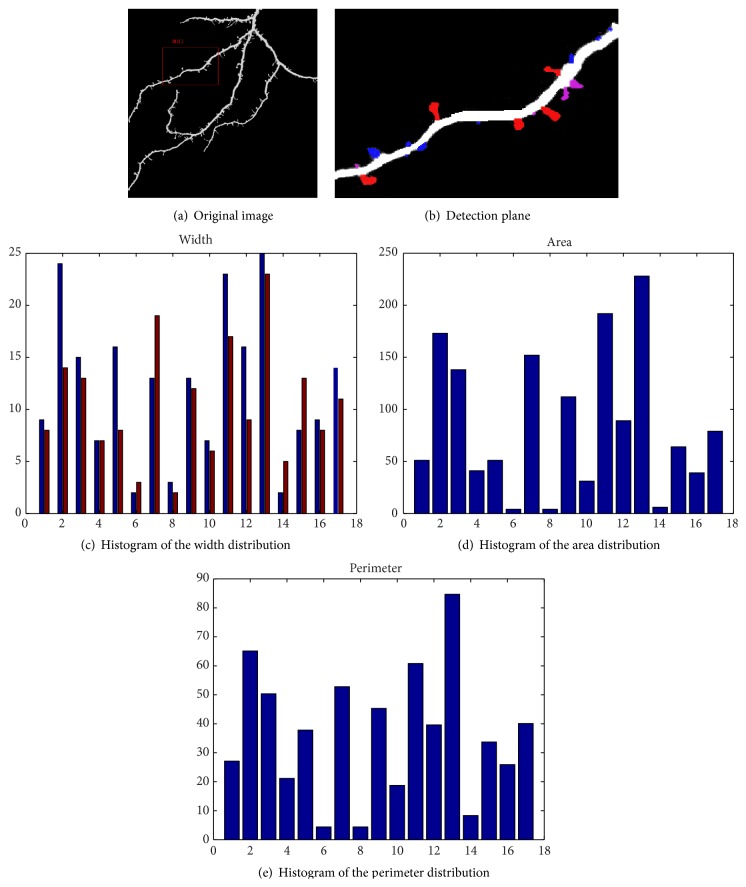
Experiment result with corresponding parameters for characterization.

Figure 8(a) shows the original image with the marked region of interest.  Figure 8(b) shows the classification result based on the features extracted in the first phase. The corresponding SE gets respect features around each pixel, but it is blind for readers to understand which features are obtained. The detected spines contain 8 mushroom types, 8 stubby types, and 4 thin types. The average of the classification accuracy of RMSNN is shown in Table 2 based on the 2700 samples in total. We can find that the detection of the mushroom and thin types has better performance than the stubby type. It is because the stubby type seems connected with the major lines, and the neck of the spine is blurred. Figures 8(c), 8(d), and 8(e) demonstrate partial geometric attributes of the spines, including the area, perimeter, and width. We found that the areas of the spines of the ROI ranged within [10, 23] and the perimeter ranged within [8, 88].

### 3.3. Optimal Parameter in SE

According to [36], unsuitable SEs will degrade the performance of the RMSNN; hence, it is critical to choose the proper SEs. According to the average size of the spines as 20 by 20 pixels, we selected SEs with different sizes and shapes to test the performance. The comparison of classification accuracies based on the 2700 samples is shown in Table 3. We can find that the disk with a radius of 4 pixels reaches the best performance. Therefore, we finally defined the SEs as a disk with the radius of 4 pixels.

### 3.4. Algorithm Comparison

**Figure 9:**
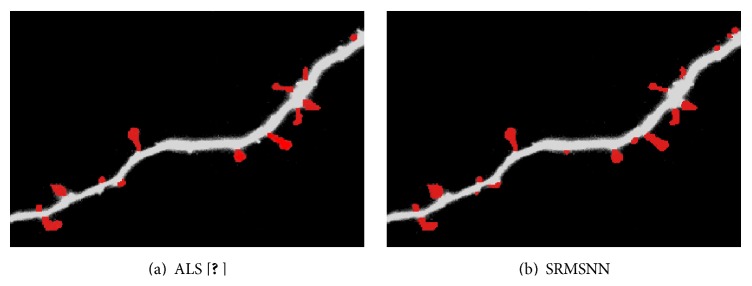
Detection result based on ALS and SRMSNN.

To further validate the efficacy of our proposed approach, we have compared the proposed algorithm with Cheng et al.'s method [18] and the manual method. In Cheng et al.'s paper, the authors employed the adaptive threshold to segment the image and Chen and Molloi's algorithm [37] to extract the backbone and then used the local SNR for the detection of the detached spine and local spine morphology for the detection of the attached spines. The comparison results based on ROI1 in Figure 8 and 15 images collected in our database are shown in Table 4. It is found from Figure 9 that Cheng et al.'s method missed some small protrusions whose number of pixels is more than 5. The number of detected spines via our algorithm is 19, 13 by Cheng et al.'s method, and 20 via the manual method as shown in Table 4. Cheng et al.'s method is robust at dealing with the spines detached from the dendrite but weak at spines attached with the dendrite. However, the detached spines from the dendrite are caused by the deconvolution to denoise the image. Our proposed algorithm overcomes the problem of detecting attached spines.

## 4. Discussion

In this paper, we have proposed new algorithms using conditional symmetric analysis and regularized morphological shared-weight neural network to detect and analyze the dendrite and dendritic spines.

Figure 5 shows that backbone extraction result based on the conditional symmetry analysis. Compared to the second-order directional derivatives method in [14], our proposed algorithms reduced the computation time of linking the breaking point of the backbone.

Figure 6 shows the result of the marked backbone and the boundary of the dendrite, which is used to determine the starting point of the spines.

Table 2 shows the classification result of the different types of spines. The row in Table 2 stands for the actual class and the column in Table 2 stands for the predicted class. The “mushroom” type has an obvious head and thin neck. The “stubby” type lacks obvious neck, and the “thin” type lacks obvious head. In Table 2, the detection accuracy of the mushroom type is higher than the other two types, and part of the stubby type is misclassified into mushroom and thin types as its head and neck ratio is at the level of average. A percent of 1.1 of thin spines are misclassified into mushroom type and 0.3% into stubby type, which is caused by the similar size of the head and neck. Table 4 shows the result of detected spines of Figure 8, respectively, by manual, ALS [18], and our proposed method SRMSNN. The results demonstrate that our algorithm has better performance than the other two methods for the images obtained by the confocal laser scanning microscopy.

## 5. Conclusion

In this paper, we proposed a new automatic approach to accurately identify dendritic spines with different shapes. The novelty of this approach includes (1) a new model using wavelet-based conditional symmetry analysis for dendrite backbone extraction and localization, which is the first step towards identification of dendritic spins; (2) a new algorithm based on regularized morphological shared-weight neural networks for classification of spines into the right classes (i.e., mushroom, stubby, and thin), entitled “RMSNN.” This research was based on our collected microscopy images. We have applied our approach to image base library containing around 2700 subimage samples, 900 for each type of spines, and have compared the proposed method with the existing methods. The experimental results demonstrate that our algorithm outperforms existing methods with a significant improvement in accuracy in terms of classifying spines into the different spine categories. The classification accuracy is 99.1% for mushroom spines, 97.6% for stubby spines, and 98.6% for thin spines.

The future work will be focusing on further validation of the robustness of the algorithms through collecting more samples and testing on different datasets. A user-friendly interface will be also built for usability improvement and enhancement. Meanwhile, we will be focusing on reducing the computation time while improving the classification accuracy based on the 3D image stacks. Other feature extraction tools (such as wavelet packet analysis [38], wavelet entropy [39], and 3D-DWT [40]) and other advanced classification tools [41, 42] will be tested. Besides, swarm intelligence method will be used to find optimal parameters [43].

## Figures and Tables

**Table 1:** Parameters of the feature extraction phase.

	Parameter	Definition
Input	*a*(*x*)	The input to a node *y* from node *x*
	*t*_*y*_(*x*)	Connections associating the node *y* with node *x*
	*t*_*y*_^*h*^(*x*_*y*_)	Hit SE associating node *y* with node *x*
	*t*_*y*_^*m*^(*x*)	Miss SE associating node *y* with *x*
	*w*_*y*_^*h*^(*x*)	Weight for Miss SE node *y* with *x*
	*w*_*y*_^*m*^(*x*)	Weight for Hit SE node *y* with *x*

Output	*a*_*y*_	The output of node *y*

**Table 2:** Average of the classification accuracy on a 10-by-10 CV.

Spine types	Mushroom	Stubby	Thin
Mushroom	99.1%	1.3%	1.1%
Stubby	0.7%	97.6%	0.3%
Thin	0.2%	1.1%	98.6%

**Table 3:** Classification accuracy by different SEs (unit is in pixel, bold denotes the best, *r* is radius, and *w* is width).

	Disk (*r* = 5)	Disk (*r* = 4)	Disk (*r* = 3)	Square (*w* = 3)	Square (*w* = 4)
Mushroom	98.7%	**99.1**%	95.4%	85.3%	89.2%
Stubby	96.2%	**97.6**%	94.1%	87.2%	91.2%
Thin	94.3%	**98.6**%	96.2%	79.1%	75.3%

**Table 4:** Detection result of ROI1 in Figure 8 and 15 images in our database.

Methods	ROI1	15 images
Manual	20	2021
ALS [18]	13	1750
SRMSNN (proposed)	19	1987
